# Particle and Cell Separation in Deterministic Lateral Displacement Arrays with Inverse L-Shaped Pillars

**DOI:** 10.3390/mi16050546

**Published:** 2025-04-30

**Authors:** Hao Jiang, Fengyang Zhang, Zhou Fan, Chundong Zhang, Zunmin Zhang

**Affiliations:** State Key Laboratory of Materials-Oriented Chemical Engineering, College of Chemical Engineering, Nanjing Tech University, Nanjing 211816, China; 202261204357@njtech.edu.cn (H.J.); zfy824254729@163.com (F.Z.); fanzhoulijiang@gmail.com (Z.F.); zhangcd@njtech.edu.cn (C.Z.)

**Keywords:** deterministic lateral displacement, dissipative particle dynamics, particle separation, cell sorting, pillar shape

## Abstract

Deterministic lateral displacement (DLD) has emerged as a powerful microfluidic technique for label-free particle separation with high resolution. Although recent innovations in pillar geometry have broadened its biomedical applications, the fundamental mechanisms dictating flow behavior and separation efficiency remain not fully understood. In this study, we conducted dissipative particle dynamics simulations to systematically investigate the separation of rigid spherical particles and red blood cells (RBCs) in DLD arrays with inverse L-shaped pillars. The simulations established a predictive formula for the critical separation size in such devices and demonstrated that inverse L-shaped pillars enabled a reduced critical separation size compared with conventional circular pillars. Additionally, we revealed that the inverse L-shaped pillars could act as deformability sensors, promoting localized RBC deformation near their protrusions and inducing stiffness-dependent bifurcation in cell trajectories, which enables effective sorting based on cell deformability. These findings advance the mechanistic understanding of inverse L-shaped DLD arrays and provide valuable design principles for their potential applications.

## 1. Introduction

Deterministic lateral displacement (DLD) has emerged as a powerful microfluidic technique for continuous label-free particle separation with exceptional resolution and versatility [1,2,3,4]. By exploiting intrinsic particle properties such as size, shape, and deformability, DLD enables precise sorting across various biomedical applications, including the isolation of rare cells (e.g., circulating tumor cells) [5,6,7], fractionation of blood components [8,9,10], and removal of pathogens from biological fluids [11,12,13]. Its robustness and compatibility with complex biofluids further highlight its potential in clinical diagnostics and therapeutic development.

**Figure 1:**
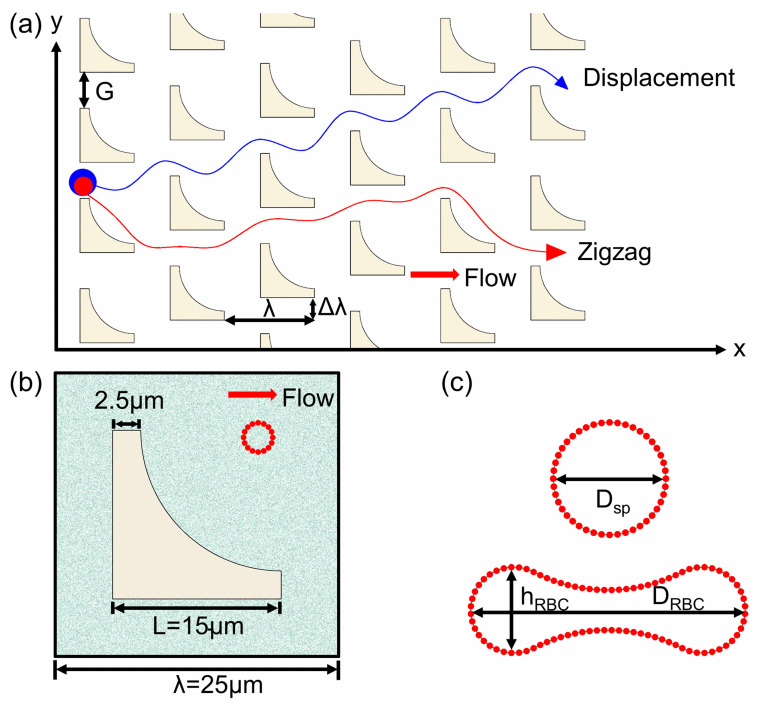
Illustrations of (**a**) DLD principle, (**b**) two-dimensional simulation system involving one inverse L-shaped pillar, one rigid spherical particle, and many fluid beads, and (**c**) DPD models of the rigid spherical particle and red blood cell.

The principle of DLD relies on the asymmetric bifurcation of laminar flow in a row-shifted pillar array. Particles larger than a critical separation size ($D_{c}$) are displaced laterally along the pillar gradient (displacement mode), while smaller particles follow the flow direction in a zigzag trajectory (zigzag mode), as illustrated in Figure 1a. A mixed mode, characterized by irregular alternations between displacement and zigzag motions, has also been experimentally and computationally observed [14,15]. The critical separation size $D_{c}$, theoretically defined as twice the width of the first flow lane adjacent to a pillar [16], is influenced by geometric parameters such as pillar shape, row shift fraction (ε=Δλ/λ), and inter-pillar gap (*G*). While empirical formulas, such as $D_{c}=1.4G\varepsilon^{0.48}$ for circular pillars, have been established to provide foundational design guidelines, their validity diminishes for deformable or anisotropic particles. Moreover, these formulas are primarily derived for symmetric or simple pillar geometries, and their dependency on complex shapes remains poorly characterized [17].

To improve sorting performance and broaden the scope of applications, significant efforts have been directed towards optimizing pillar geometries and array layouts to address limitations inherent in conventional designs [18,19,20]. For instance, airfoil-shaped pillars have been demonstrated to significantly reduce hydrodynamic resistance, thereby minimizing cell deformation and clogging during separation processes [21,22]. Numerical simulations suggested that their streamlined shape could maintain a stable velocity gradient, effectively reducing shear stress on soft cells. Additionally, I-shaped pillars have been developed to enhance the sorting efficiency of non-spherical bioparticles, such as red blood cells (RBCs), by inducing controlled rotational dynamics that leverage their largest dimension as the effective separation parameter [23]. Furthermore, triangular pillars with sharp edges have been proven to facilitate the deformability-based sorting by promoting cell bending around these edges [24]. Collectively, these advancements highlight the critical role of pillar geometry in tailoring flow–particle interactions, enabling precise control over separation mechanisms for diverse biomedical applications.

Among these novel designs, the L-shaped and inverse L-shaped pillars exhibit unique advantages for cell sorting due to their pronounced geometric asymmetry, characterized by groove and protrusion structures that disrupt flow symmetry and induce rotational and deformational dynamics for non-spherical particles [18]. In particular, the inverse L-shaped design has demonstrated strong potential for deformability-based cell separation, with successful applications in enriching human reticulocytes from erythroid culture [25], profiling white blood cells for infection diagnostics [26], and isolating mesenchymal stem cells from bone marrow aspirates [27]. Despite these promising results, the fundamental mechanisms governing their sorting performance remain poorly understood, particularly the complex interaction between pillar geometry, flow modulation, and cell deformation.

To address this gap, we employ dissipative particle dynamics (DPD) simulations to systematically investigate the separation of rigid spherical particles and red blood cells (RBCs) within inverse L-shaped pillar arrays. The particle trajectories, flow field modulation, and cell deformation dynamics have been carefully studied to elucidate the underlying sorting mechanisms. These findings are expected to provide foundational insights for the design of novel pillar geometries and the advancement of inverse L-shaped DLD devices in complex bioparticle sorting applications.

## 2. Simulation Method and Models

### 2.1. Dissipative Particle Dynamics Simulation

DPD is a mesoscopic particle-based computational approach, enabling efficient simulations of complex hydrodynamic phenomena [28]. In the method, the fluid and suspended particles are represented as coarse-grained beads interacting through three pairwise forces: a conservative force ($F_{ij}^{C}$) derived from a soft repulsion potential, a dissipative force ($F_{ij}^{D}$) proportional to relative velocities, and a random force ($F_{ij}^{R}$) accounting for thermal fluctuations. These forces are defined as [29]:

(1) $$F_{ij}^{C}=a_{ij}\left(1-r_{ij}/r_{c}\right){\hat{r}}_{ij}$$

(2) $$F_{ij}^{D}=-\gamma \omega^{D}\left(r_{ij}\right)\left(v_{ij}\cdot {\hat{r}}_{ij}\right){\hat{r}}_{ij}$$

(3) $$F_{ij}^{R}=\sigma \omega^{R}\left(r_{ij}\right)\theta_{ij}{∆t}^{-1/2}{\hat{r}}_{ij}$$

where $r_{ij}$ is the inter-bead distance, $r_{c}$ is the cutoff radius, $v_{ij}$ is the relative velocity, and ∆t is the time step.$\;a_{ij}$ is the repulsion parameter between two beads i and j, γ is the friction coefficient, and σ is the noise amplitude. $\theta_{ij}$ is a random number with a zero average and unit variance. $\omega^{D}\left(r_{ij}\right)$ and $\omega^{R}\left(r_{ij}\right)$ are the distance-dependent weight functions for the dissipative and random forces, respectively. A common form of the weight function is given by

(4) $$\omega^{R}\left(r_{ij}\right)=\left\{\begin{array}{cc} {\left(1-r_{ij}/r_{c}\right)}^{p}, & r_{ij}<r_{c}\\ 0, & r_{ij}\geq r_{c} \end{array}\right.$$

To obey the fluctuation–dissipation theorem and ensure system equilibrium at temperature *T*, $\omega^{D}\left(r_{ij}\right)={\left[\omega^{R}\left(r_{ij}\right)\right]}^{2}$ and $\sigma^{2}=2\gamma k_{B}T$ should be satisfied. In addition, p=0.15, $r_{c}=1.5$, and ∆t=0.002 were selected based on our previous study [15].

### 2.2. Models and Simulation Settings

In this study, we employed a two-dimensional simulation framework with a constant bead number density of *n* = 5, incorporating a single suspended rigid spherical particle, a stationary inverse L-shaped pillar, and fluid particles within the computational domain. As depicted in Figure 1b, the inverse L-shaped pillars were designed by subtracting a quarter-circle section (radius = 12.5 μm) from a square pillar with a side length of L = 15 μm, which matches the experimental design reported by Ranjan et al. [18]. It is worth noting that the pillar is composed of frozen DPD beads, maintaining an equilibrium structure identical to that of the surrounding fluid. In addition, the fluid particles are represented by individual DPD beads, while the suspended rigid spherical particles and RBCs are modeled as closed bead–spring chains (Figure 1c). The spring potential is defined as:

(5) $$V_{sp}=\sum_{j\in 1\ldots N_{p}}\left[\frac{k_{B}Tl_{m}\left(3x_{j}^{2}-2x_{j}^{3}\right)}{4p\left(1-x_{j}\right)}+\frac{k_{p}}{l_{j}}\right]$$

where $k_{p}$ is the spring constant, $l_{j}$ is the length of the spring j, $l_{m}$ is the maximum spring extension, $x_{j}=l_{j}/l_{m}\in (0,1)$, and p is the persistence length. Note that the balance between the two force terms in Equation (5) leads to a nonzero equilibrium spring length $l_{0}$ with $l_{m}/l_{0}=2.2$. In addition, a combination of a bending potential and an area constraint potential is incorporated to induce a 2D biconcave shape for the cell and to maintain the rigid spherical shape of the suspended particles in equilibrium. The specific forms of these potentials are given as follows:

(6) $$U_{bend}=\sum_{j=1}^{N_{p}}k_{b}\left[1-\cos\left(\theta_{j}\right)\right]$$

(7) $$U_{area}=k_{a}\frac{{\left(A-A_{0}\right)}^{2}}{2}$$

where $k_{b}$ and $k_{a}$ denote the bending and area constraint coefficients, respectively. $\theta_{j}$ represents the instantaneous angle between two adjacent bonds. A is the instantaneous area, and $A_{0}$ denotes its target area.

In this work, five rigid spherical particles with diameters of $D_{sp}$ = 2.74 μm, 3.20 μm, 3.65 μm, 4.10 μm, and 4.56 μm were constructed to evaluate the size-based separation performance of the inverse L-shaped pillar arrays. For RBCs, the bending coefficient $k_{b}$ in 2D is related to the macroscopic bending rigidity *κ* by the relation $\kappa =k_{b}l_{0}$. A typical healthy RBC has an effective 2D diameter $D_{r}^{2D}=6.1$ μm, and a bending rigidity *κ* of approximately 50–70$k_{B}T$ at a physiological temperature of T = 37 °C. The bending rigidity of RBCs is further characterized by a dimensionless rigidity factor $K_{2D}^{*}=\kappa /\kappa_{0}$, where the reference bending rigidity $\kappa_{0}/k_{B}Tl_{0}=50$ is a healthy RBC. Accordingly, five types of RBCs with varying bending rigidity, $K_{2D}^{*}$ = 1, 10, 20, 40, and 60, are modeled to evaluate the capability of deformability-based RBC sorting. For additional details regarding the models and parameters, please refer to our previous works [15,17,24,30].

All simulations were performed using the Large-scale Atomic/Molecular Massively Parallel Simulator (LAMMPS) [31]. Periodic boundary conditions were applied along both the flow (x) and lateral (y) directions to model an infinite array of inverse L-shaped pillars. To capture the characteristic row-shifted configuration of DLD systems, a lateral offset (row shift, Δ*λ*) was introduced in the y direction whenever a DPD bead traversed the periodic boundary along the x direction. In this study, the fluid flow in the x direction was driven by a uniform external force applied to each DPD bead, simulating a pressure-driven system under experimental conditions and maintaining a steady laminar flow. In addition, an additional force in the y direction was introduced to ensure no net flow in the lateral direction [15]. At the solid–fluid interface, a bounce-back reflection scheme was implemented for both the fluid and suspended particles, preventing wall penetration and ensuring no-slip boundary conditions. Moreover, an adaptive tangential shear force was applied within a near-wall region of thickness, $r_{c}$, further stabilizing the velocity profile near the surface [15].

## 3. Results and Discussion

### 3.1. Size-Based Particle Separation

**Figure 2:**
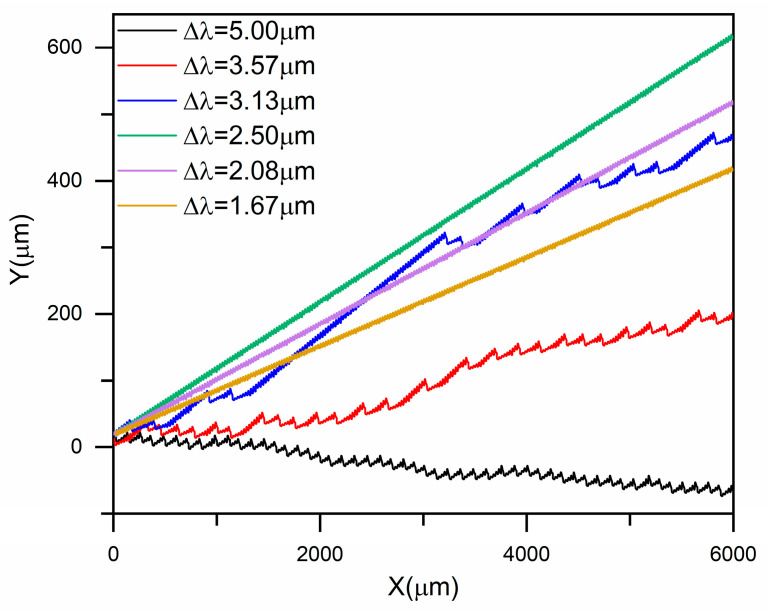
Trajectory curves of a representative rigid spherical particle of size $D_{sp}=4.10$ μm in inverse L-shaped pillar arrays under different row shifts.

**Figure 3:**
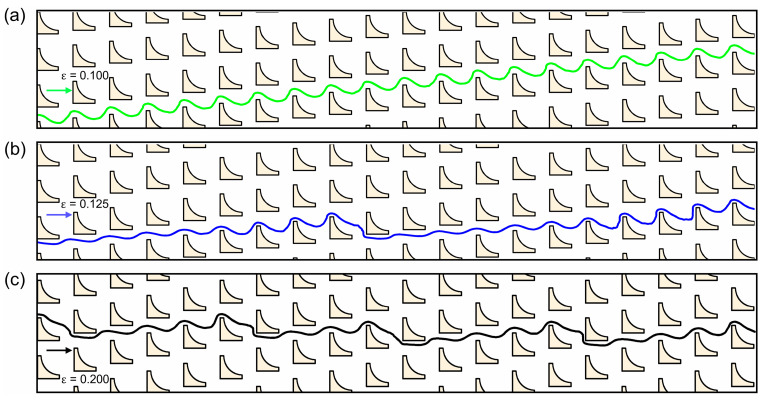
Illustration of the typical trajectories of particles with $D_{sp}=4.10$ μm in inverse L-shaped pillar arrays under different row shift fractions (ε=∆λ/λ): (**a**) 0.100, (**b**) 0.125, and (**c**) 0.200.

The size-dependent separation behavior of rigid spherical particles was systematically investigated in inverse L-shaped pillar arrays by varying both the particle diameter particles ($D_{sp}=2.74\sim 4.56$ μm) and the row shift fraction (ε=Δλ/λ). Figure 2 presents the representative trajectories of a $D_{sp}=4.10$ μm particle across different DLD arrays. Under small row shifts (Δλ≤2.50 μm), the particle exhibited a linear trajectory with consistent lateral migration, corresponding to the displacement mode, where it moved along the pillar gradient (Figure 3a). At intermediate row shifts (e.g., Δλ≤3.13 μm), the particle trajectories became irregular (Figure 3b), suggesting a mixed mode. When the row shift was further increased to Δλ≥3.57 μm, the particle exhibited a clear zigzag path (Figure 3c), indicative of the zigzag mode in which particles followed the average flow direction. This progressive transition from displacement to zigzag mode, passing through a mixed regime as the row shift increases, aligns with the classical principle of DLD separation.

In order to quantitatively assess the particle traversal behavior in a DLD array, we defined a dimensionless parameter, “separation index” ($I_{s}$), which is the ratio between the average lateral displacement of a particle per pillar and the row shift Δ*λ* [15]. A value of $I_{s}=1.0$ represents an ideal displacement mode, where larger particles shift laterally at each pillar. On the other hand, $I_{s}=0.0$ indicates an ideal zigzag mode, where smaller particles experience no net lateral movement. For particles of intermediate size, a mixed mode is commonly observed, characterized by irregular zigzagging periods, and this particle size is often used to define the critical separation size in DLD devices at particular row shifts. In this study, we considered $I_{s}$ values within the range of [0.3, 0.6] as indicative of the mixed mode and the transition between displacement and zigzag behavior. Therefore, $I_{s}>0.6$ and $I_{s}<0.3$ were used to represent the displacement and zigzag modes, respectively.

**Figure 4:**
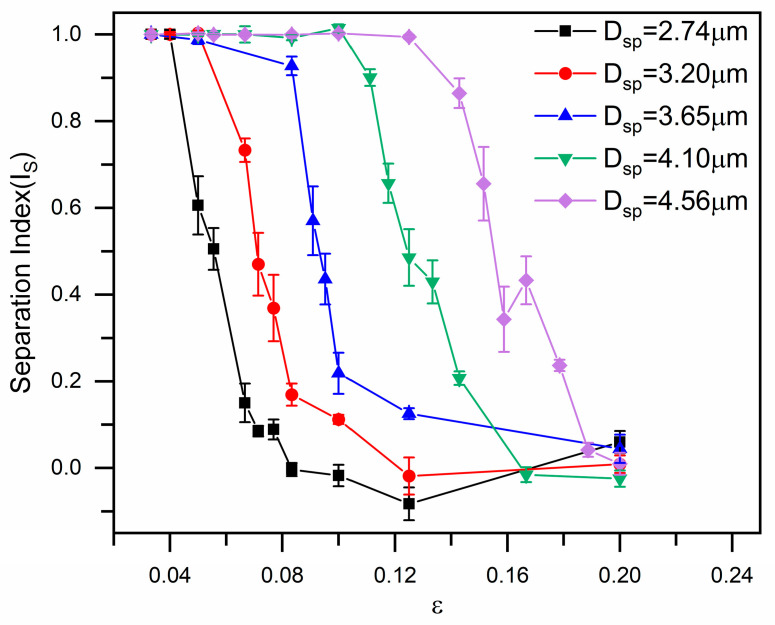
Separation index ($I_{s}$) for various rigid spherical particles in inverse L-shaped pillar arrays as a function of the row shift fraction.

To further explore the mode transition boundaries, we applied the same analytical framework to quantitatively evaluate the separation of all five particle diameters within inverse L-shaped pillar arrays. Figure 4 summarizes the measured separation indices for each particle size as a function of the row shift fraction, revealing a clear size-dependent trend in the displacement–zigzag mode transition. It is apparent that the mixed mode regime progressively shifted toward higher values of the row shift fraction with increasing the particle diameter, consistent with the DLD principle that a larger row shift fraction leads to a greater critical separation size. For example, the 2.74 μm particle transitioned from displacement to zigzag mode at *ε* = 0.056, whereas the 3.20 μm particle exhibited this transition at *ε* = 0.071.

According to our previous study [15], a generalized formula was proposed to calculate the critical separation size of DLD arrays with various pillar geometries:

(8) $$D_{c}=\alpha G\varepsilon^{\beta }$$

where the dimensionless parameters *α* and *β* are determined by the shape and spatial arrangement of pillars. For the inverse L-shaped design investigated in this study, nonlinear regression yielded optimal values of *α* = 1.08 and *β* = 0.48, leading to

(9) $$D_{c}=1.08G\varepsilon^{0.48}$$

This derived equation provides a practical guideline for the design and application of inverse L-shaped DLD devices, enabling precise tuning of structural parameters such as the gap size and row shift fraction to meet specific separation requirements. Nevertheless, the applicability of the derived formula is limited to the specific pillar geometry considered in this study. Changes in the dimensions of the groove structure are expected to significantly affect both the critical separation size and the form of the derived formula.

In comparison with the conventional circular pillar design ($D_{c}=1.4G\varepsilon^{0.48}$), the critical separation diameter of inverse L-shaped DLD devices is significantly smaller, which aligns well with the experimental observations reported by Ranjan et al. [18]. According to DLD theory, such differences in separation performance are primarily attributed to the geometry-induced modifications of the flow field within the array, which can be analytically characterized by evaluating the width of the first flow lane [16].

**Figure 5:**
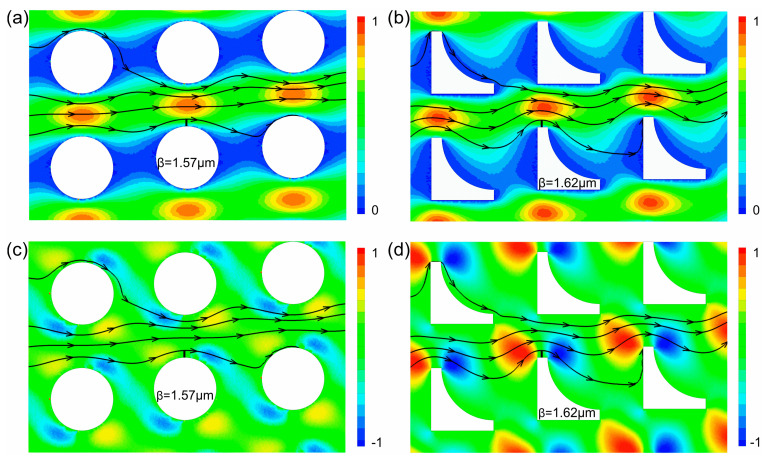
Simulated flow profiles of circular and inverse L-shaped pillar arrays with *ε* = 0.100: (**a**,**b**) x-component of velocity along the flow direction; (**c**,**d**) y-component of velocity perpendicular to the flow direction. Representative simulated streamlines are plotted as black solid lines with arrows.

To better understand the influence of pillar geometry on separation behavior, we numerically analyzed the fluid flow fields around circular and inverse L-shaped pillars under identical row shift fractions. Stall lines were mapped to quantify the first flow lane width in each case (Figure 5). Notably, the inverse L-shaped pillars exhibited a slightly wider first flow lane (1.62 μm) compared with the circular counterparts (1.57 μm), as indicated by the x-component velocity profiles in Figure 5a,b. This observation appears counterintuitive, given the smaller critical separation size associated with the inverse L-shaped design. Ranjan et al. [18] suggested that a sudden expansion or contraction of fluid streams caused by grooved pillar geometries might induce abrupt changes in particle translational momentum, thereby generating inertial forces acting on the particles. To further investigate this effect, we analyzed the corresponding velocity profiles in the y-direction, which was perpendicular to the primary flow direction. As shown in Figure 5c,d, the inverse L-shaped pillars induced two pronounced upward and downward flow regions near the pillar protrusions, which were substantially stronger than those observed in the circular pillar design. These flow features may facilitate particle escape from the original streamlines, promote transverse migration, and result in lateral displacement across flow lanes.

### 3.2. Deformability-Based Cell Sorting

Our previous study demonstrated that sharp-edged pillar geometries can act as deformability sensors by inducing RBC bending around the edges, enabling the deformability-based sorting of RBCs [24]. According to this concept, we hypothesized that the protrusion of the inverse L-shaped pillar, whose dimensions were significantly smaller than the RBC diameter, might similarly promote localized membrane deformation and thereby enhance sensitivity to differences in cell deformability. To test this, we quantified RBC deformation using the acircularity parameter *δ*_2*D*_ as cells traversed the inverse L-shaped pillar array. The acircularity *δ*_2*D*_ is defined as

(10) $$\delta_{2D}=\frac{{\left(\lambda_{1}-\lambda_{2}\right)}^{2}}{{\left(\lambda_{1}+\lambda_{2}\right)}^{2}}$$

where $\lambda_{1}$ and $\lambda_{2}$ are the square roots of the eigenvalues of the 2D radius-of-gyration tensor. This parameter characterizes the deviation of a cell’s projected shape from a perfect circle, with *δ*_2*D*_ = 0.0 corresponding to a circular shape and values approaching 1.0 indicating highly elongated geometries. At equilibrium, typical 2D RBCs exhibit *δ*_2*D*_ = 0.29, consistent with their biconcave shape.

**Figure 6:**
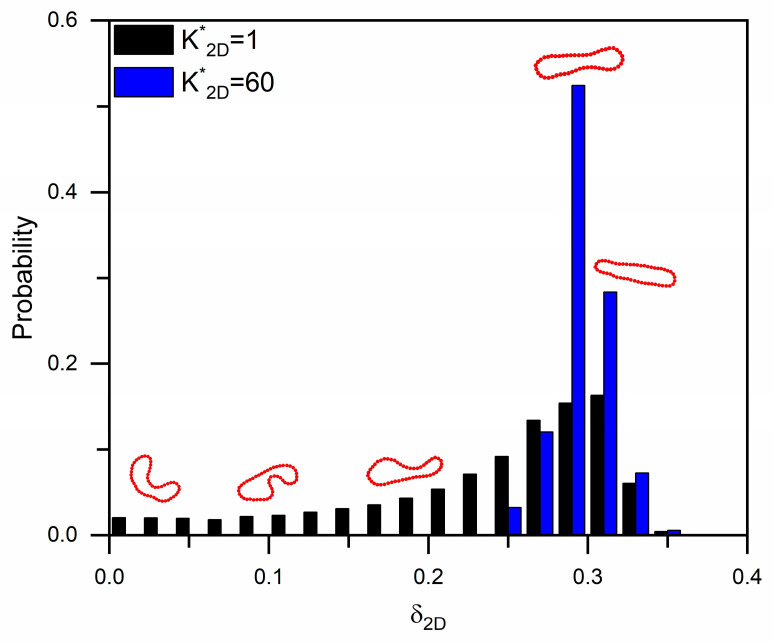
Acircularity distributions of soft and stiff RBCs flowing in inverse L-shaped pillar arrays with *ε* = 0.125.

Figure 6 presents the acircularity distributions of soft ($K_{2D}^{*}=1$) and stiff ($K_{2D}^{*}=60$) RBCs as they traverse inverse L-shaped pillar arrays at *ε* = 0.125. Compared with the circular pillars [24], the inverse L-shaped design induces significantly greater cell deformation, particularly for the more deformable RBCs. These results suggest that the sharp structural features of the inverse L-shaped pillars can amplify cell deformation and may contribute to improved sorting resolution based on mechanical properties.

**Figure 7:**
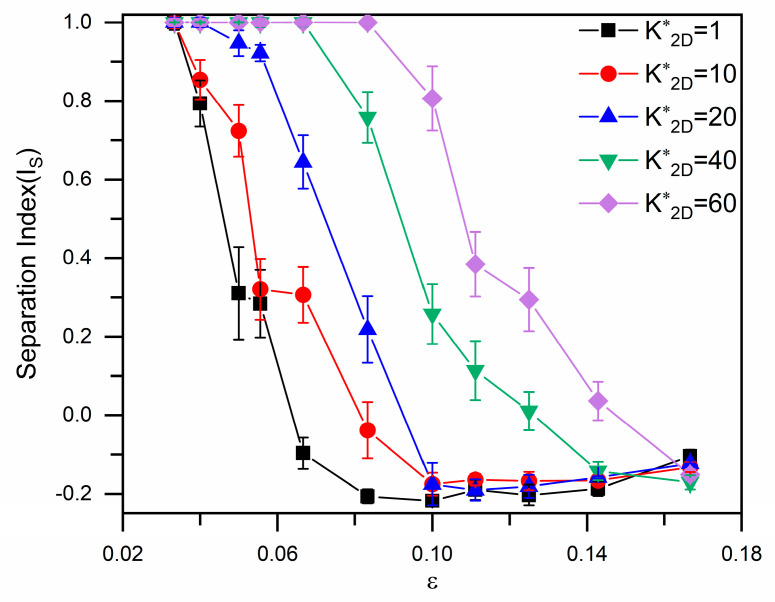
Separation index ($I_{s}$) for different RBCs in inverse L-shaped pillar arrays as a function of the row shift fraction.

Figure 7 presents the separation index for RBCs traversing the inverse L-shaped pillar arrays as a function of the row shift fraction and cell bending rigidity. In contrast with the minimal dependence of the displacement-to-zigzag transition on RBC stiffness observed in circular pillar arrays [24], this transition exhibits a marked sensitivity to bending rigidity in the inverse L-shaped pillar design. As shown in Figure 7, the critical row shift fraction at which the transition occurs systematically increases with RBC bending rigidity, indicating that stiffer cells require a larger row shift to transition from displacement to zigzag mode.

**Figure 8:**
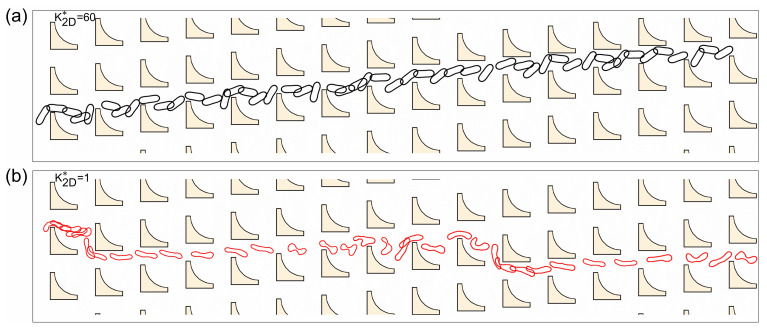
Illustration of the cell trajectories of (**a**) stiff and (**b**) soft RBCs in inverse L-shaped pillar arrays with *ε* = 0.083.

To gain further insight into the underlying mechanism of such deformability-dependent transitions, we examined representative trajectories and deformation behaviors of RBCs with different bending rigidities. At a row shift fraction of *ε* = 0.083, Figure 8 illustrates the traversal paths of soft ($K_{2D}^{*}=60$) and stiff ($K_{2D}^{*}=1$) RBCs in the inverse L-shaped pillar array. Notably, the stiff RBC follows a displacement trajectory, while the soft RBC predominantly exhibits a zigzag motion. This distinct divergence in cell trajectories is evidently attributed to differences in deformation. As seen in Figure 8, the stiff RBC undergoes minimal shape change and is repeatedly bumped by the pillar protrusions, whereas the soft RBC deforms significantly while traversing the array, particularly upon interacting with the protruding corners of the pillars.

**Figure 9:**
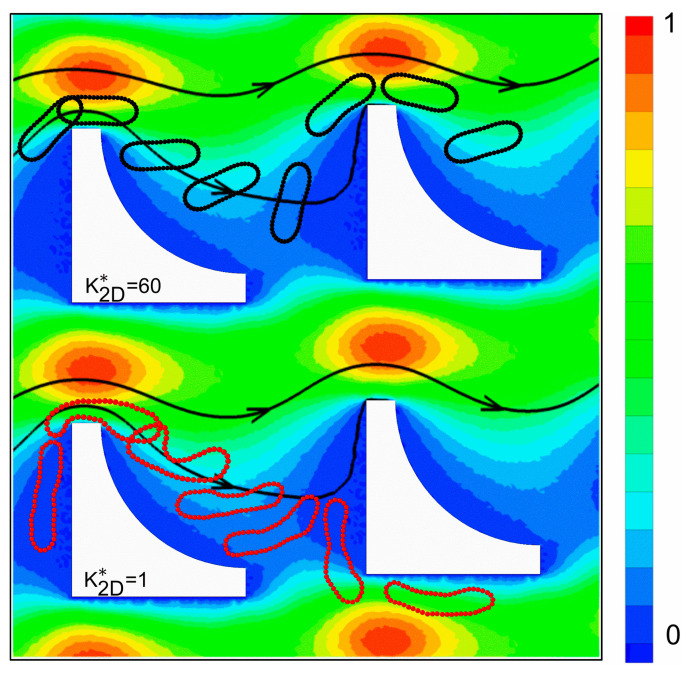
Snapshots of the movement and deformation of soft and stiff RBCs in inverse L-shaped pillar arrays with *ε* = 0.083.

For a closer examination of the underlying deformation dynamics, Figure 9 presents representative snapshots of RBC morphology and movement near an individual inverse L-shaped pillar. The stiff RBC keeps its elongated shape with negligible deformation. Upon encountering the pillar protrusion, its rigid structure prevents it from adapting to the local flow path, effectively extending its projected length beyond the first streamline. Consequently, it is repeatedly bumped away from the pillar and undergoes lateral displacement. In contrast, the soft RBC undergoes pronounced shape deformation, especially at the pillar protrusions. At these locations, the cell exhibits substantial bending and occasional flipping motions, which enable it to conform to the surrounding flow field and remain within the first flow lane, ultimately resulting in a zigzag trajectory. This deformability-induced morphological adaptation underlies the observed trajectory bifurcation and underscores the critical role of localized cell–pillar interactions in mediating deformability-dependent transitions.

This rigidity-dependent bifurcation in motion is consistent with the deformability-based sorting mechanism previously observed in triangular pillar geometries, where sharp structural features promote bending-induced sorting [24]. In the present case, the inverse L-shaped protrusion plays a similar role by functioning as a deformability-sensitive filter: only cells with sufficiently low bending rigidity can undergo the necessary shape deformation to remain within the streamline. The observed sorting sensitivity arises not only from differences in deformation amplitude but also from the interaction between the effective cell length and the flow stream length along the direction of motion. Specifically, a deformable cell that bends around the protrusion effectively reduces its projected length, allowing it to stay within the primary flow lane. In contrast, a rigid cell, unable to conform to the pillar geometry, exceeds the stream length and is laterally displaced.

This mechanism represents a distinct departure from classical size-based separation, introducing a deformability-based criterion governed by localized cell–pillar interactions. It further reinforces the concept that only spatially confined flow-induced deformations near pillar protrusions can significantly influence traversal behavior, a key insight also supported by previous studies on sharp-edged geometries [24]. These findings demonstrate that inverse L-shaped pillars serve not only as passive obstacles but as active modulators of cell deformation and trajectory, thereby offering a promising design for DLD devices aimed at sorting bioparticles based on subtle mechanical heterogeneity.

## 4. Conclusions

In this study, we employed DPD simulations to systematically investigate the size- and deformability-based separation behaviors of rigid spherical particles and red blood cells (RBCs) in DLD arrays with inverse L-shaped pillars. The simulation results demonstrated that the inverse L-shaped geometry enabled a reduced critical separation size compared with conventional circular pillars, owing to its unique anisotropic shape that modulates the local flow field and promotes transverse particle migration. A predictive formula for the critical separation size was established specifically for the inverse L-shaped design, offering a practical guideline for tuning structural parameters such as row shift fraction and gap size to meet specific separation requirements. However, it was evident that the dimensions and positioning of the groove structure in the inverse L-shaped pillar could significantly influence the critical separation size and the applicability of the predictive formula. These design parameters, such as groove size and location, should be considered in future studies to develop a generalized formula that can guide the design and application of various L-shaped pillar arrays.

Furthermore, we revealed that the inverse L-shaped pillars could act as effective deformability sensors, amplifying the differences in RBC mechanical properties through localized cell deformation near their protrusions. By coupling geometric asymmetry with cell deformation, these pillars could induce a stiffness-dependent bifurcation in cell trajectories, establishing a clear and controllable mechanism for deformability-based cell sorting.

In summary, these findings advance the mechanistic understanding of how pillar geometry governs microfluidic particle separation and highlight the potential of the inverse L-shaped pillar arrays as a versatile and tunable platform for high-resolution bioparticle sorting. Building on these insights, our study offers general design guidelines for DLD device optimization, which may facilitate the development of microfluidic systems tailored to selectively target intrinsic cell properties such as size and deformability.

## Data Availability

All original data presented in this study are included within the article. Additional details are available from the corresponding author upon reasonable request.

## Abbreviations

The following abbreviations are used in this manuscript:

DLD	Deterministic Lateral Displacement
DPD	Dissipative Particle Dynamics
RBC	Red Blood Cell
